# Epigenetic therapy attenuates oxidative stress in BMSCs during ageing

**DOI:** 10.1111/jcmm.17089

**Published:** 2021-12-07

**Authors:** Xiaoxia Su, Haoyu Zhang, Fengzhen Lei, Rui Wang, Tingting Lin, Li Liao

**Affiliations:** ^1^ State Key Laboratory of Oral Diseases & National Clinical Research Center for Oral Diseases & Department of Pediatric Dentistry & Engineering Research Center of Oral Translational Medicine & National Engineering Laboratory for Oral Regenerative Medicine West China Hospital of Stomatology Sichuan University Chengdu China; ^2^ Key Laboratory of Shaanxi Province for Craniofacial Precision Medicine Research Department of Orthodontics College of Stomatology Xi’an Jiaotong University Xi'an China

**Keywords:** bone remodelling, cell differentiation, epigenetics, oxidative stress, stem cells

## Abstract

Oxidative stress, a hallmark of ageing, inhibits the osteogenic differentiation of bone marrow‐derived mesenchymal stem cells in long bone. The dysfunction of the cellular antioxidant defence system is a critical cause of oxidative stress, but the mechanism of the decline of antioxidant defence in senescent stem cells remains elusive. Here, we found that EZH2, an epigenetic regulator of histone methylation, acted as a suppressor of the antioxidative defence system in BMSCs from the femur. The increased EZH2 led to a decrease in the levels of antioxidant enzymes and exaggerated oxidative damage in aged BMSCs, resulting in the defect of bone formation and regeneration. Mechanistically, EZH2 enhanced the modification of H3K27me3 on the promoter of Foxo1 and suppressed its function to activate the downstream genes in antioxidant defence. Moreover, epigenetic therapy targeting EZH2‐mediated H3K27me3 modification largely recovered the antioxidant defence in BMSCs and attenuate oxidative damage, leading to the recovery of the osteogenesis in old BMSCs. Taken together, our findings revealed novel crosstalk between histone epigenetic modification and oxidative stress during stem cell ageing, suggesting a possibility of epigenetic therapy in the recovery of BMSCs senescence and treatment of age‐related bone disease.

## 1 INTRODUCTION

Ageing usually leads to the decline of strength and destruction of the skeletal structure. As recorded, the peak bone mass declines since the age of 30.^1^ About half of females and one‐fifth of males older than 50 years will suffer fragility osteoporosis in the remaining lifetime.^2^ Along with the destruction of micro‐architecture, the regeneration of bone also declines along with ageing, which affects the repair of bone defects and fractures.^3^ Bone marrow‐derived mesenchymal stem cells (BMSCs), which are the source of the progenitor of pre‐osteoblasts, are essential in bone formation and remodelling of the skeleton.^4^ However, ageing is a key etiological factor of primary osteoporosis. Ageing usually led to the senescence of BMSCs, resulting in specific senescent phenotypes and reduced capacities of bone formation and regeneration.^5^, ^6^, ^7^ How to promote bone formation in the aged skeleton is a critical issue in the clinic.

Oxidative stress, which is enhanced during ageing, plays a central role in the senescence of stem cells.^8^ Physiologically, cells possess an intracellular system to eliminate redundant reactive oxygen species (ROS) and prevent oxidative damage.^9^ Several regulators of the antioxidative defence system will be activated by redundant reactive oxygen species (ROS) to activate the expression of a series of antioxidant enzymes, such as superoxide dismutase (Sod) and catalase (Cat).^10^ During ageing, the antioxidant defence system failed to protect the stem cells from oxidative damage.^11^ Reactivation of antioxidant defence is a possible strategy to recover rejuvenate senescent stem cells.

Emerging evidence suggests that aberrant epigenetic modifications on histone are hallmarks of ageing and closely related to ageing.^12^, ^13^ Methylation on lysine of histone 3 is one of the essential epigenetic mechanisms that control gene expression.^14^, ^15^ Methylation on histone has been demonstrated to modulate the lineage differentiation and function of BMSCs.^16^ Importantly, several specific epigenetic modifications on histone are changed during ageing, leading to aberrant expression of key transcription factors or signalling molecules.^14^, ^17^, ^18^ Multiple epigenetic regulators have been identified as drivers of cellular senescence and are the potential therapeutic target of age‐related diseases.^19^ Since the epigenetic modifications are reversible, it is possible to prevent or even rescued stem cell ageing by targeting key epigenetic regulators.^20^ A remarkable example comes from the research showing that deletion of components of histone methylation complexes for H3K27 extends lifespan in flies,^21^ suggesting epigenetic therapy might be a promising strategy to suppress cellular senescence. Thanks to the development of novel epigenetic drugs, several epigenetic therapies have been applied in the treatment of tumours.^22^ Moreover, emerging evidence supports that epigenetic drugs could have important roles in synergy with other traditional therapies,^23^ suggesting the huge potential of epigenetic drugs in the clinic.

In our previous study, we found that enhancer of Zeste Homolog 2 (EZH2), a methyltransferase, was increased in BMSCs during osteoporosis, resulting in the suppression of osteogenic differentiation of BMSCs.^24^ Interestingly, we found by chance that BMSCs overexpressed EZH2 also express higher levels of ROS, while knockdown of EZH2 leads to a decrease in ROS levels in senescent BMSCs, suggesting a connection between epigenetic regulation and the oxidative stress in stem cell ageing. Therefore, in this study, we moved forward to investigate whether EZH2 attributes to the oxidative stress in BMSCs during ageing and explore novel epigenetic therapy to rejuvenate senescent BMSCs.

## 2 MATERIALS AND METHODS

### 2.1 Animals

This study is an animal study following the ARRIVE Guidelines. All animal procedures were performed according to the guidelines of the Animal Care Committee of the Sichuan University, Chengdu, China. 2‐month‐old and 20‐month‐old male C57BL/6J mice and 2‐month‐old male nude mice were purchased from the Dashuo Bio Company (Chengdu, China) and housed under specific pathogen‐free conditions (22℃, 12‐hour light/12‐hour dark cycles and 50–55% humidity) with free access to food pellets and tap water during the study. Based on the data of our previous studies, at least 5 mice were allocated to each group in the animal experiments. There are no criteria for inclusion and exclusion of samples.

### 2.2 Cell culture

Bone marrow‐derived mesenchymal stem cells were cultured as previously reported.^25^ In brief, the femurs of mice were collected and the attached soft tissues were removed. Bone marrow cavities of the femur were flushed with a‐MEM (Invitrogen; Carlsbad, CA, USA) supplemented with 10% foetal bovine serum (FBS), 1% penicillin and streptomycin. The cell suspension was filtrated with 70‐μm cell strainers, and the isolated cells were seeded in 10‐cm tissue culture dishes and cultured with α‐MEM growth medium (Invitrogen; Carlsbad, CA, USA) containing 10% FBS (Sijiqing; Hangzhou, China), 1% penicillin and streptomycin. The dishes were placed in a humidified atmosphere of 5% CO_2_ at 37℃, and the medium was changed every 2–3 days to remove non‐adherent cells. When the adherent cells were confluent, BMSCs were passaged after digestion with 0.25% trypsin/1 mM ethylene diamine tetraacetic acid (EDTA). BMSCs at 2~3 passages were used in the experiments.

### 2.3 SA‐β Gal staining

Senescence‐associated beta‐galactosidase (SA‐β Gal) staining was performed using a commercial staining kit according to standard protocol. In brief, BMSCs are fixed with paraformaldehyde, washed in PBS, and stained with freshly prepared SA‐staining solution. After staining, BMSCs were washed again in PBS. These cells stained blue‐green were considered as SA‐β‐Gal‐positive cells. The ratio of these SA‐β Gal^+^ to total cells was counted manually in the images taken under microscopy.

### 2.4 Osteogenic differentiation in vitro

In vitro osteogenic differentiation and analysis were performed as we previously reported. BMSCs were cultured in an osteogenic induction medium containing 100 μg/ml ascorbic acid, 2 mM b‐glycerophosphate and 10 nM dexamethasone. The medium was changed every 3 days. An ALP staining kit was used to detect ALP activity in BMSCs 7 days after induction. The formation of mineralized nodules was detected with alizarin red staining 14 days after induction.

### 2.5 Detection of intracellular ROS levels

To detect the intracellular ROS levels in BMSCs, 25 mM 20,70‐dichlorofluorescein diacetate (DCFH‐DA) (Genmed, Shanghai, China) were added into the culture medium and incubated for hr in darkness. The fluorescence of 2’,7'‐dichlorofluorescein was measured by using a fluorescent microscope or flow cytometry.

### 2.6 Real‐time RT‐PCR

Total RNA was isolated after cell lysis with Trizol reagent. mRNA was reverse transcribed into cDNA using a kit. The expression of mRNA was detected with the SYBR‐Green method using a Bio‐Raid PCR machine. The primers used in this study were listed in Supplemental Table S1.

### 2.7 ChIP analysis

Chromatin immunoprecipitation (CHIP) analysis was performed with a Chromatin Immunoprecipitation Kit (Millipore) according to the standard protocol as previously reported.^11^, ^24^ In brief, the chromatin fragments derived from BMSCs of young or aged mice were immunoprecipitated with 6 μg of antibody against EZH2 and H3K27me3 (Santa Cruz). DNA was extracted, and real‐time PCR analysis was performed for the promoter of Foxo1.

### 2.8 Immunofluorescence assay of intracellular FoxO1

Immunofluorescence assay was performed to detect the subcellular localization of FoxO1 in BMSCs as those described previously.^11^ The primary antibody for FoxO1 was purchased from Cell Signaling Technology. The samples were examined under a confocal microscope (Olympus Optical; Tokyo, Japan).

### 2.9 Western blot analysis

Western blot analysis was performed as previously reported.^25^ Antibodies used in this study were anti‐EZH2, anti‐H3K27me3, anti‐GAPDH, anti‐FOXO1 and anti‐β‐actin.

### 2.10 HE staining of cell transplants

The transplants of ectopic bone formation were fixed by paraformaldehyde for 24 hr and then demineralized with 10% EDTA for 4 weeks. The transplants were embedded in paraffin and sectioned at 5 mm. HE staining was performed according to standard protocols.

### 2.11 Ectopic bone formation assay

To evaluate the osteogenic capacity of BMSCs in vivo, 5 × 10^6^ BMSCs combined with 50 mg hydroxyapatite/tricalcium phosphate (HA/TCP) were transplanted subcutaneously into the dorsal of nude mice. The nude mice were randomly divided into two groups. Three days after transplantation, 100 μg/kg 3‐deazaneplanocin A (DZNep) (Merck Millipore, Cat No.252790) or an equal amount of dimethyl sulphoxide (DMSO) (as vehicle control) was injected subcutaneously around the transplants every 3 days for 6 weeks. The transplants were then harvested for histological analysis.

### 2.12 Statistical analysis

Data are reported as means ± SD. Results were analysed using SPSS 16.0 software (SPSS Inc., Chicago, IL, USA). Continuous data (mean ± SD) that follow a normal distribution were compared using Student's *t* test where two groups were compared and analysed using parametric or non‐parametric analysis of variance (ANOVA) where more than two groups were compared. Significance was confirmed at *p *< 0.05.

## 3 RESULTS

### 3.1 Defect of antioxidant defence leads to decreased osteogenic differentiation of senescent BMSCs

**FIGURE 1:**
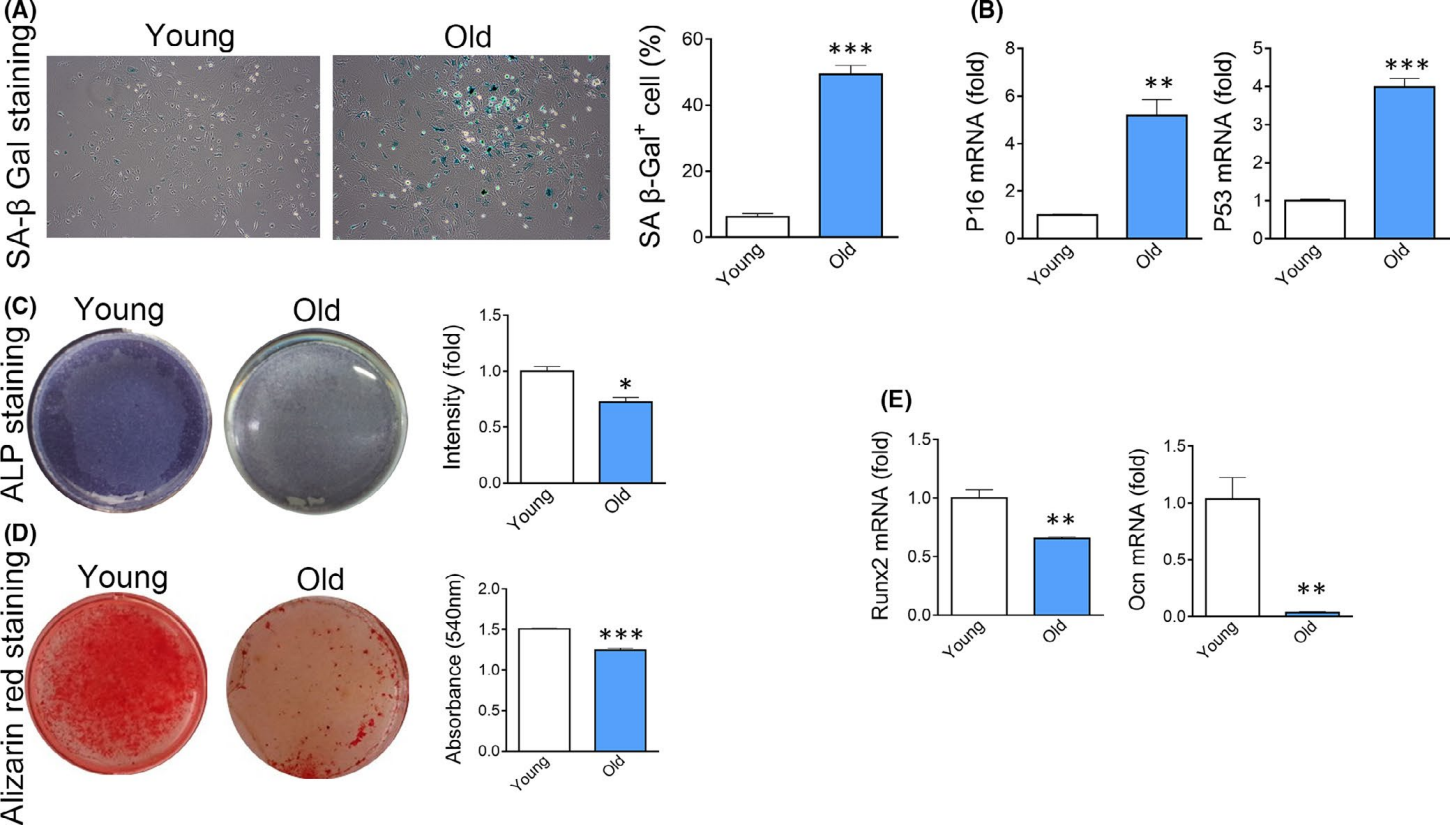
Senescence of BMSCs derived from old mice. (A) Expression of SA β‐Gal in BMSCs derived from young or old mice. *n *= 5. (*t* test) (B) Real‐time analysis of senescence marker gene P16^INK4a^ and P53 in BMSCs derived from young or old mice. *n *= 3. (*t* test) (C) ALP staining of BMSCs derived from young or old mice after 7 days of osteogenic induction. *n *= 4. (*t* test) (D) Alizarin red staining of BMSCs derived from young or old mice after 14 days of osteogenic induction. *n *= 4. (*t* test) (E) Expression of Runx2 and Ocn in BMSCs derived from young or old mice after 14 days of osteogenic induction. *n *= 3. (*t* test) Data are shown as mean ± SD. *p < 0.05, **p < 0.01, ***p < 0.001. NS, no significance. *n*, the number of biological replicated

We adopted 20‐month‐old mice as an age‐related osteoporosis model in this study (Figure S1A, S1B). BMSCs isolated from the femur of aged mice highly expressed senescence markers including senescence‐associated β‐Gal (SA β‐Gal) (Figure 1A), P16^INK4a^ and P53 (Figure 1B). In vitro osteogenic assay showed that BMSCs derived from aged mice (Old BMSCs) expressed less ALP (Figure 1C) and formed less mineralized nodules (Figure 1D) after osteogenic induction, comparing with young BMSCs. Real‐time RT‐PCR revealed a decrease in the levels of Ocn and Runx2, two master genes controlling osteogenesis, in old BMSCs (Figure 1E). These results demonstrated that BMSCs suffer cellular senescence during ageing.

**FIGURE 2:**
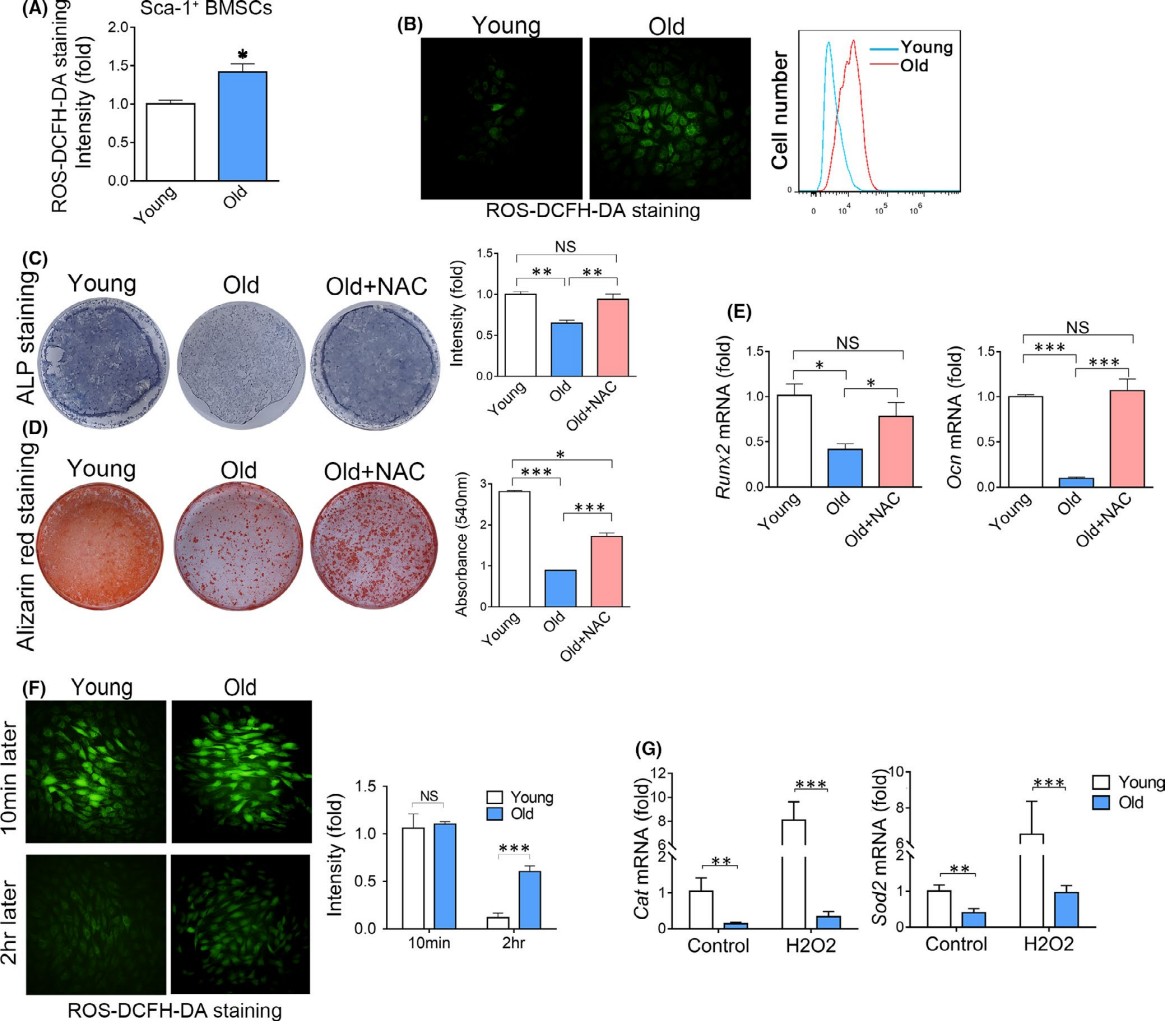
Osteogenic differentiation of BMSCs is suppressed by redundant oxidative stress. (A) Flow cytometry analysis of ROS levels in Sca‐1^+^ bone marrow cells derived from the femurs of young or old mice. *n *= 3. (*t* test) (B) The ROS in BMSCs derived from young or old mice were detected under a fluorescent microscope (left) and FCM (right). (C) ALP staining of BMSCs treated with or without NAC after 7 days of osteogenic induction. *n *= 3. (*t* test) (D) Alizarin red staining of BMSCs treated with or without NAC after 14 days of osteogenic induction. *n *= 3. (*t* test) (E) Real‐time RT‐PCR analysis of Runx2 and Ocn in BMSCs treated with or without NAC after 14 days of osteogenic induction. *n* =4. (ANOVA) (F) Young and old BMSCs were treated with H_2_O_2_. The ROS levels in BMSCs 10 min and 2 hr after treatment were analysed by fluorescent microscope and FCM. *n* = 4. (ANOVA) (G) Real‐time RT‐PCR analysis of Cat and Sod2 in young and old BMSCs before and after treatment of H2O2. *n* = 3. (ANOVA) Data are shown as mean ± SD. *p < 0.05, **p < 0.01, ***p < 0.001. NS, no significance

As expected, flow cytometry (FCM) detected an increase in ROS levels in Sca‐1^+^ BMSCs in the bone marrow of aged mice (Figure 2A). To exclude the effect of microenvironment on intracellular oxidative stress, we evaluated ROS levels in young and old BMSCs cultured in the same medium for 3 passages in vitro. The ROS levels in old BMSCs remained higher than that in young BMSCs after in vitro culture (Figure 2B), suggesting a cell‐autonomous manner of oxidative stress. To confirm the effect of redundant oxidative stress on osteogenic differentiation of old BMSCs, we eliminated ROS with N‐acetylcysteine (NAC), a wildly used antioxidant. As expected, elimination of ROS by NAC partly enhanced the osteogenic differentiation of senescent BMSCs, as shown by ALP staining (Figure 2C), alizarin red staining (Figure 2D) and real‐time RT‐PCR (Figure 2E).

To explore the mechanism of oxidative stress in aged BMSCs, we added exogenous ROS into culture BMSCs and measured the elimination rate of ROS. FCM showed that old BMSCs were inefficient in eliminating exogenous ROS when compared with young BMSCs (Figure 2F), suggesting that the antioxidant defence system was inhibited during ageing. To confirm the hypothesis, we measured the levels of *Sod2* and *Cat*, two master genes in antioxidant defence. Real‐time RT‐PCR showed the levels of *Cat* decreased by 80% while the levels of *Sod2* decreased by 55% in old BMSCs, comparing with young BMSCs (Figure 2G). Importantly, after adding exogenous ROS, *Cat* and *Sod2* levels were increased by more than sixfold in young BMSCs, while exogenous ROS only induced a mild increase in *Cat* and *Sod2* in old BMSCs (Figure 2G), indicating the defect of the antioxidant defence system in BMSCs during ageing.

### 3.2 EZH2 suppresses antioxidant defence in BMSCs in aged mice

**FIGURE 3:**
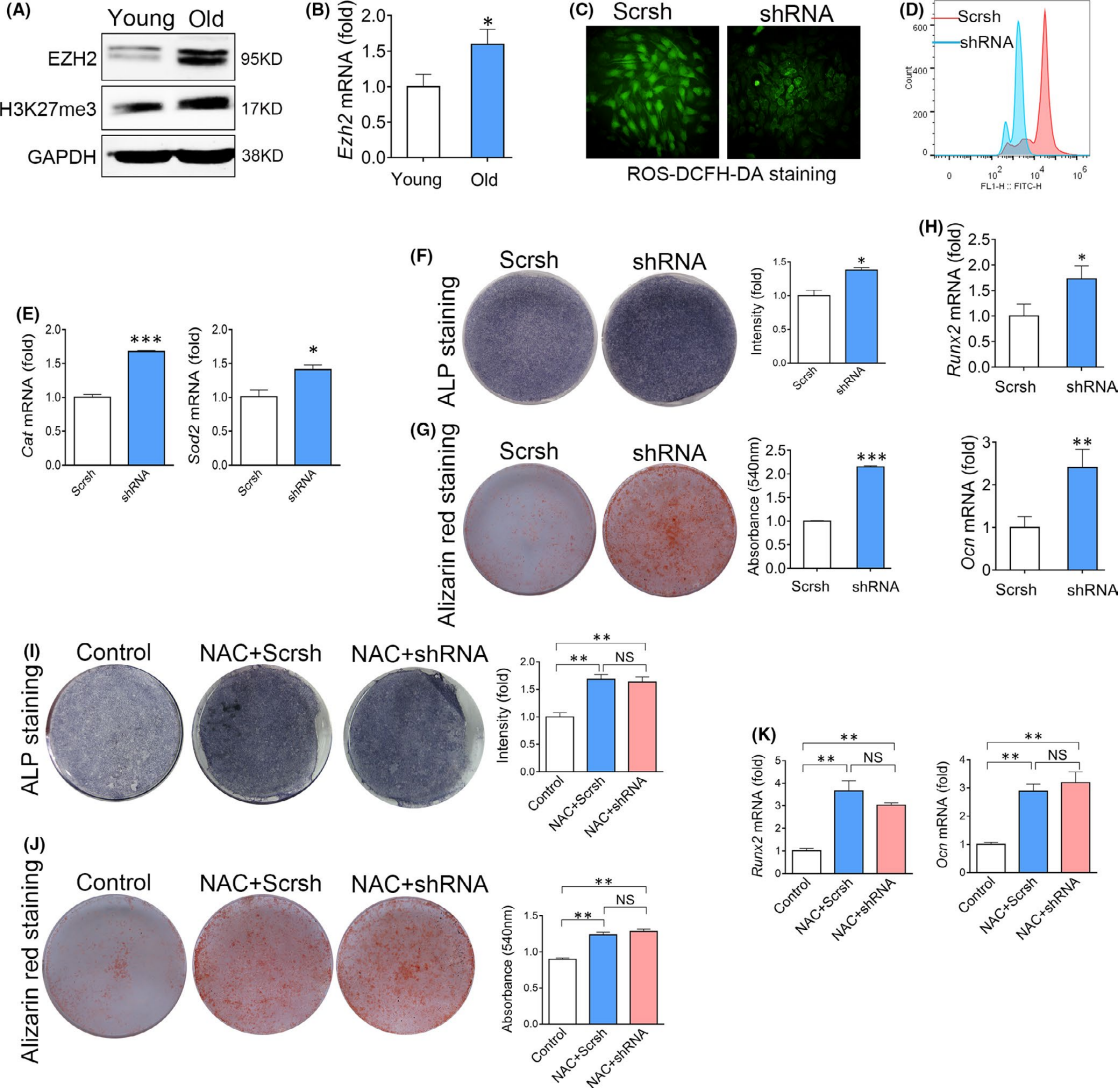
Enhancer of Zeste Homolog 2 enhances oxidative stress to suppress the osteogenic differentiation of BMSCs. (A) Western blot analysis of EZH2 and H3K27me3 levels in young or old BMSCs. (B) Real‐time RT‐PCR analysis of Ezh2 expression in young or old BMSCs. *n*=3. (*t* test) (C) Fluorescence analysis of ROS levels in BMSCs transfected with scrambled shRNA (Scrsh) or EZH2 targeting shRNA for 48hr. (D) Flow cytometry of ROS levels in BMSCs transfected with EZH2 shRNA or Scrsh. (E) Real‐time RT‐PCR analysis of Cat and Sod2 expression in BMSCs transfected with EZH2 shRNA or Scrsh. *n *= 3. (*t* test) (F) Alp staining of BMSCs transfected with EZH2 shRNA or Scrsh after 7 days of osteogenic induction. *n *= 3. (*t* test) (G) Alp staining of BMSCs transfected with EZH2 shRNA or Scrsh after 7 days of osteogenic induction. *n *= 3. (*t* test) (H) Real‐time RT‐PCR analysis of mRNA levels of Runx2 and Ocn in BMSCs transfected with EZH2 shRNA or Scrsh. *n *= 3. (*t* test) (I) ALP staining of BMSCs transfected with EZH2 shRNA or Scrsh after treating with NAC. *n* = 3. (ANOVA) (J) Alizarin red staining of BMSCs transfected with EZH2 shRNA or Scrsh after treating with NAC. *n* = 3. (ANOVA) (K) Real‐time RT‐PCR analysis of Runx2 and Ocn expression in BMSCs transfected with EZH2 shRNA or Scrsh after treating with NAC. *n* =3. (ANOVA) Data are shown as mean ± SD. *p < 0.05, **p < 0.01, ***p < 0.001. NS, no significance

In a previous study of EZH2, we found by chance that knockdown of EZH2 seems to decrease ROS levels in BMSCs derived from osteoporotic mice. The preliminary findings encouraged us to investigate the role of EZH2 in ROS regulation. We first confirmed that EZH2 was increased in old BMSCs compared with young BMSCs (Figure 3A). The levels of trimethylation on lysine 27 on histone 3 (H3K27me3), an important target of EZH2, were also enhanced in old BMSCs (Figure 3B). We then tested whether redundant EZH2 exaggerates oxidative stress in old BMSCs. Knockdown of EZH2 expression by shRNA resulted in a decrease in ROS levels (Figure 3C) and an increase in Cat and Sod2 levels (Figure 3D) in old BMSCs. Moreover, knockdown of EZH2 also resulted in a recovery of osteogenic differentiation of old BMSCs (Figure 3E‐G).

To further confirm that knockdown of EZH2 recovered osteogenic differentiation of old BMSCs through ameliorating ROS stress, we treated old BMSCs knocked down of EZH2 with NAC. As expected, after the elimination of ROS by NAC, knockdown of EZH2 could not further promote the osteogenic differentiation of old BMSCs (Figure 3H‐K).

### 3.3 EZH2 suppresses the transcription of Foxo1 to inhibit antioxidant defence

We then moved on to explore the mechanism of EZH2 to suppress the antioxidative system during ageing. Since EZH2 silences gene expression majorly by adding trimethylation on lysine 27 on histone 3 (H3K27me3) on the promoters of target genes, we performed a CHIP assay to analyse the binding of EZH2 on promoters of Cat and Sod2. However, we found no evidence of specific binding of EZH2 and H3K27me3 on the promoter of *Cat* and *Sod2* (Supplemental Figure S2A‐D), suggesting that EZH2 do not directly regulate their expression.

**FIGURE 4:**
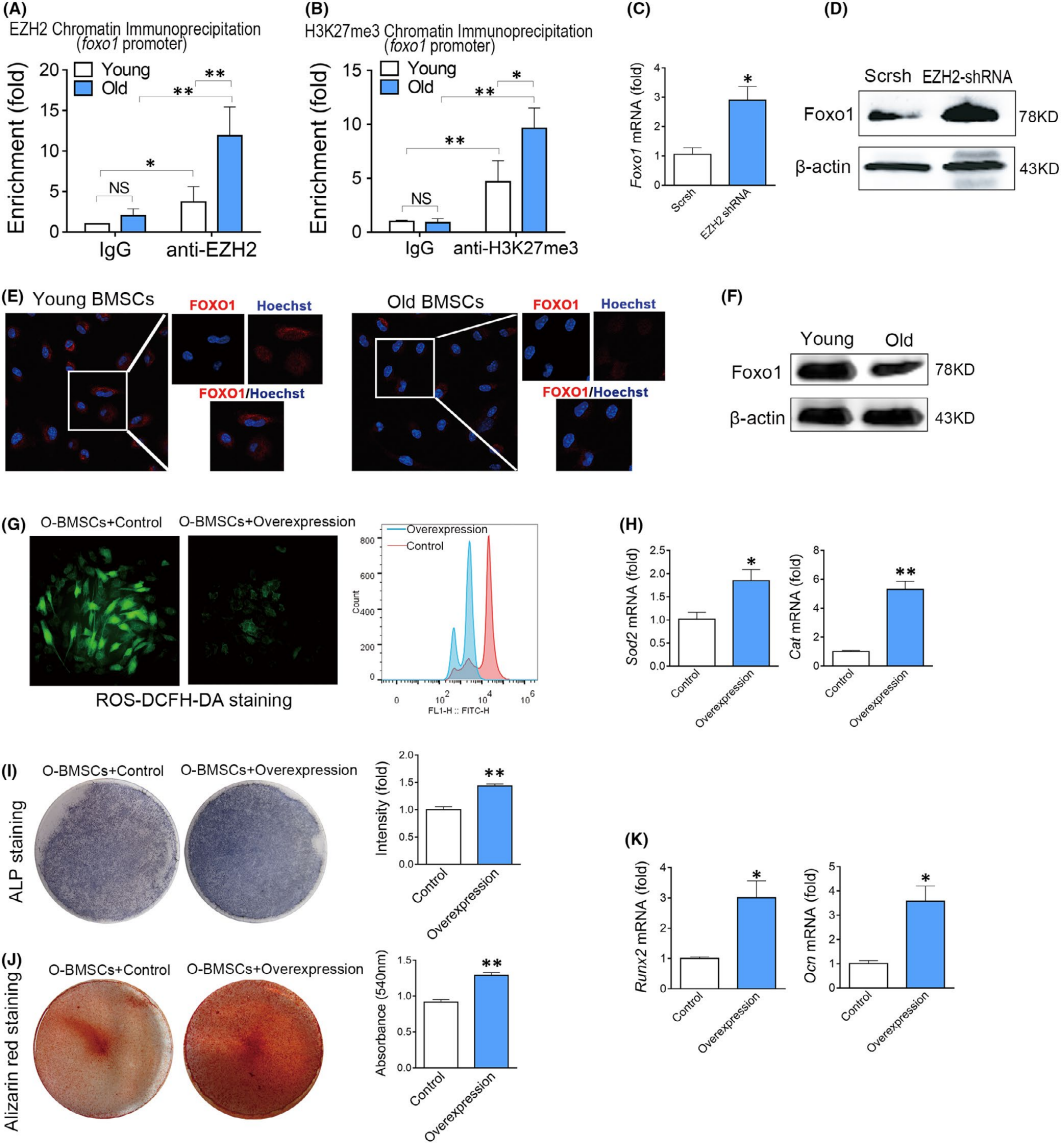
Enhancer of Zeste Homolog 2 suppresses Foxo1‐mediated antioxidant defence. (A) CHIP analysis of the binding of EZH2 at the promoter region of the Foxo1 gene. *n* = 4. (ANOVA) (B) CHIP analysis of the binding of H3K27me3 at the promoter region of the Foxo1 gene. *n* = 4. (ANOVA) (C) Real‐time RT‐PCR analysis of Foxo1 in BMSCs transfected with EZH2 shRNA or Scrsh for 48 hr. *n *= 3. (*t* test) (D) Western blot analysis of Foxo1 in BMSCs transfected with EZH2 shRNA or Scrsh for 48 hr. (E) Immunofluorescence assay of Foxo1 expression in young or old BMSCs. (F) Western blot analysis of Foxo1 expression in young or old BMSCs. (G) Fluorescence analysis of ROS levels in BMSCs transfected with Foxo1‐lentivirus or negative control. (H) Real‐time RT‐PCR analysis of Sod2 and Cat mRNA levels in BMSCs transfected with Foxo1‐lentivirus or negative control. *n *= 3. (*t* test) (I) ALP staining of BMSCs transfected with Foxo1‐lentivirus or negative control after 7 days of osteogenic induction. *n *= 3. (*t* test) (J) Alizarin red staining of BMSCs transfected with Foxo1‐lentivirus or negative control after 14 days of osteogenic induction. *n *= 3. (*t* test) (K) Real‐time RT‐PCR analysis of Runx2 and Ocn mRNA levels in BMSCs transfected with Foxo1‐lentivirus or negative control after 14 days of osteogenic induction. *n *= 3. (*t* test) Data are shown as mean ± SD. *p < 0.05, **p < 0.01, ***p < 0.001. NS, no significance

Therefore, we focused on the molecules that could regulate Cat and Sod1. Increasing evidence has demonstrated that forkhead box O (FOXO) family, especially Foxo1 and Foxo3, are crucial regulators of antioxidant defence, and closely related to the lifespan of different species.^10^, ^26^ CHIP analysis found that EZH2 could target the promoter of *Foxo1* (Figure 4A) but not *Foxo3* (data not shown). Accordingly, H3K27me3 modifications were increased in the Foxo1 promoter (Figure 4B). Knockdown of EZH2 increased the levels of *Foxo1* mRNA (Figure 4C) and proteins (Figure 4D) levels in aged BMSCs.

To confirm the role of Foxo1 in the decline of antioxidant defence in old BMSCs, we firstly compared the expression of Foxo1 in young and old BMSCs. Real‐time RT‐PCR showed that the expression of Foxo1 was obviously decreased in old BMSCs when comparing with young BMSCs. We also observed a decrease of Foxo1 in old BMSCs by immunofluorescence assay (Figure 4E) and Western blotting (Figure 4F). To confirm that the decrease of Foxo1 contributed to the defect of the antioxidant defence system in old BMSCs, we overexpressed *Foxo1* in old BMSCs using lentivirus. As expected, overexpression of Foxo1 in aged BMSCs decreased the ROS levels (Figure 4G), recovered the expression of *Cat* and *Sod2* (Figure 4H) and promoted osteogenic differentiation (Figure 4I‐K) of aged BMSCs. Taken together, these results indicated that Foxo1 is a candidate target of EZH2 to regulate antioxidant defence.

### 3.4 Inhibition of EZH2 using DZNep recovers oxidative stress‐induced osteogenic defect of old BMSCs

**FIGURE 5:**
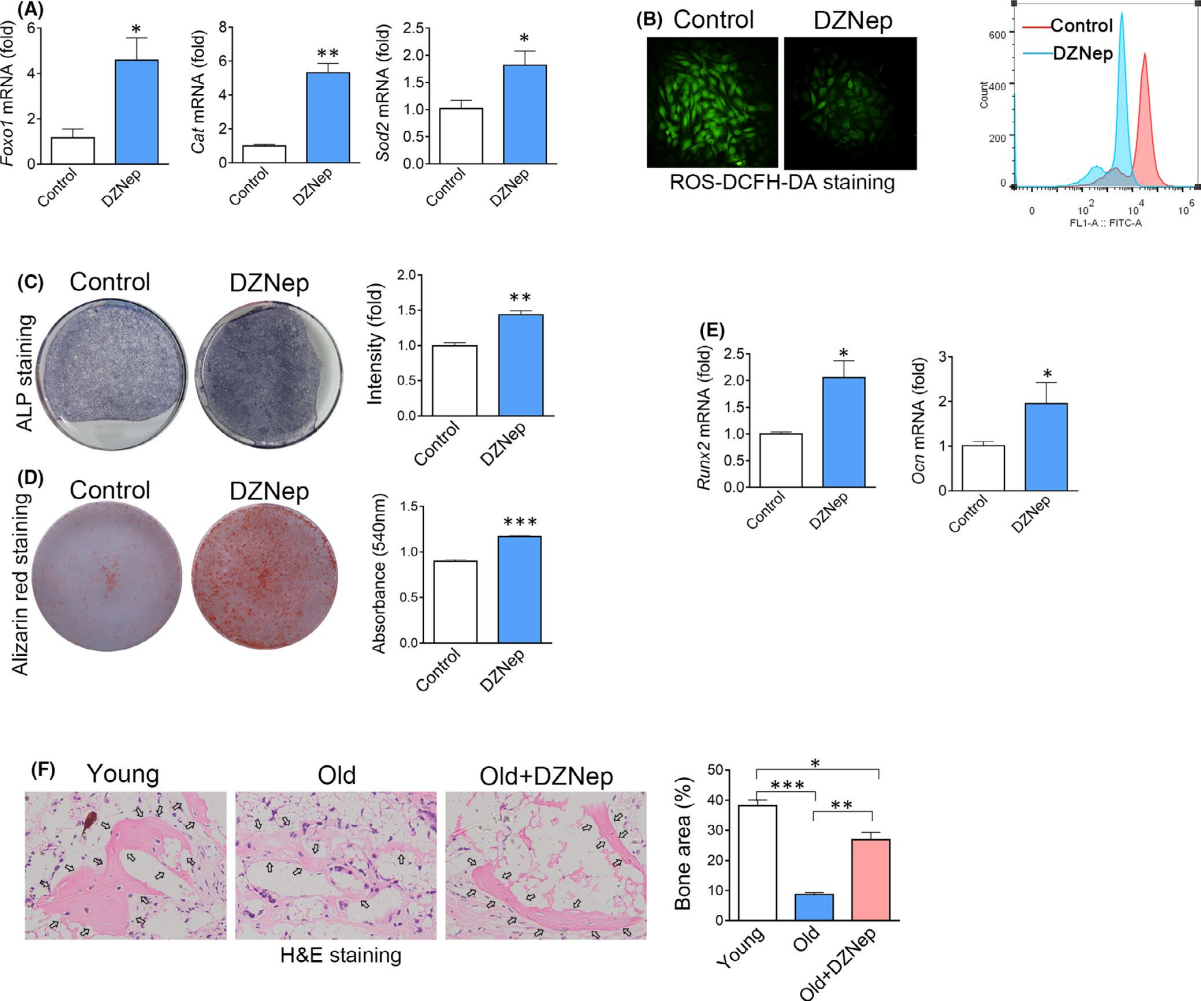
Inhibition of EZH2 recovers the osteogenic differentiation capacity of old BMSCs. (A) Real‐time RT‐PCR of Foxo1, Cat and Sod2 expression in old BMSCs treated with DMSO (as the vehicle control) or DZNep, a small molecule blocking the function of EZH2. (B) Fluorescence assay and FCM analysis of ROS in old BMSCs treated with DZNep or DMSO. (C) (I) ALP staining of old BMSCs treated with DZNep or DMSO after 7 days of osteogenic induction. *n *=3. (*t* test) (D) Alizarin red staining of old BMSCs treated with DZNep or DMSO after 14 days of osteogenic induction. *n *= 3. (*t* test) (E) Real‐time RT‐PCR analysis of Runx2 and Ocn mRNA levels in old BMSCs treated with DZNep or DMSO after 14 days of osteogenic induction. *n* = 3. (*t* test) (F) Histological analysis of transplants of young BMSCs or old BMSCs treated with DMSO or DZNep after 1month of subcutaneous transplantation. *n* = 4. (ANOVA) Data are shown as mean ± SD. *p < 0.05, **p < 0.01, ***p < 0.001. NS, no significance. *n*, the number of biological replicated

**FIGURE 6:**
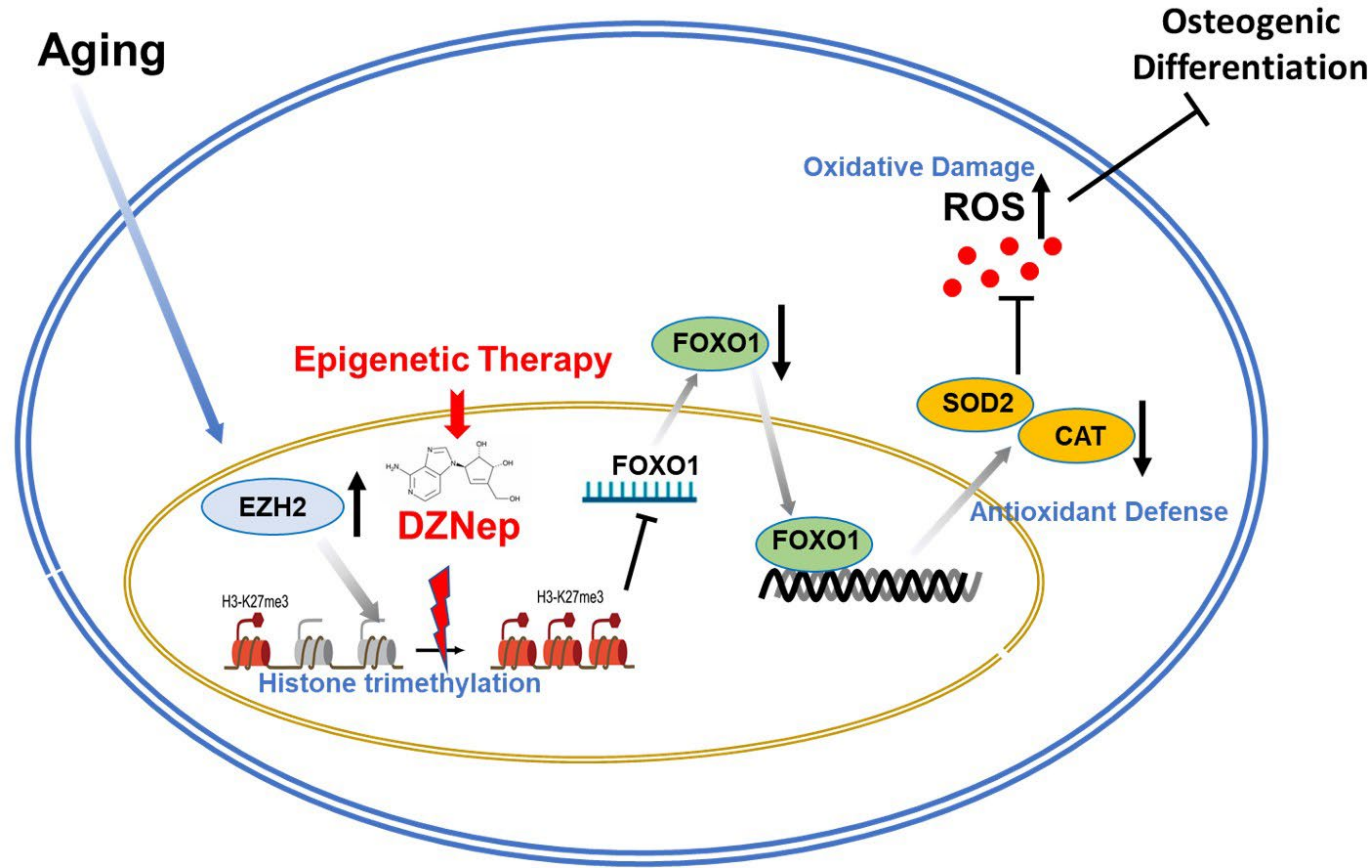
Graphic abstract of the study. EZH2 enhanced the modification of H3K27me3 on the promoter of Foxo1 and suppressed its function to activate the downstream genes in antioxidant defence. The increased EZH2 led to a decrease in the levels of antioxidant enzymes and exaggerated oxidative damage in aged BMSCs, resulting in the defect of bone formation and regeneration. Epigenetic therapy targeting EZH2‐mediated H3K27me3 modification largely recovered the antioxidant defence in BMSCs and attenuated oxidative damage, leading to the recovery of the osteogenesis in old BMSCs

Lastly, we tried to rescue the dysfunction of senescent BMSCs using a small molecule that inhibiting EZH2 function. DZNep, a reagent widely used to block the methylation process, was used as an inhibitor of EZH2. Treatment of DZNep resulted in increased Foxo1 mRNA in aged BMSCs (Figure 5A). We also observed an increase of CAT and Sod1 expression and a decrease in ROS levels in aged BMSCs after treatment of DZNep (Figure 5A,B). We then detected the in vitro osteogenic differentiation of old BMSCs treated with DZNep. ALP staining, alizarin red staining and real‐time RT‐PCR analysis showed that DZNep efficiently recovered the osteogenic differentiation of old BMSCs in vitro (Figure 5C‐E). To further confirm the function of DZNep, we transplanted the old BMSCs pretreated with DZNep with HA/TCP into the subcutaneous of nude mice for ectopic bone formation. Three months after transplantation, the histological analysis confirmed that young BMSCs formed abundant mineralized bone matrix, while old BMSCs formed much less mineralized bone matrix. Notably, DZNep treatment largely recovered the osteogenesis capacity of old BMSCs in vivo (Figure 5F). Taken together, these results showed that epigenetic therapy targeting EZH2 recovers the osteogenic differentiation capacity of senescent BMSCs (Figure 6).

## 4 DISCUSSION

Oxidative stress and aberrant epigenetic modification are two hallmarks of ageing.^8^ In this study, we found that these mechanisms are closely related. The increase of trimethylation on histone eventually led to the decline of antioxidant defence in BMSCs, which contribute to the dysfunction of senescent BMSCs. Mechanistically, EZH2‐enhanced trimethylation on H3K27 in the promotor of Foxo1 to inhibit the activation of the antioxidative defence system. By blocking the methylation using small molecules would re‐activate Foxo1‐mediated antioxidant defence and alleviated the decline of osteogenesis in ageing BMSCs. Taken together, our findings uncovered a novel link between epigenetic modification and oxidative stress in stem cell ageing, suggesting a novel target to alleviate oxidative‐induced stem cell ageing.

Enhancer of Zeste Homolog 2 has been demonstrated to suppress the osteogenic differentiation of normal BMSCs. The mechanism of EZH2 is versatile, including transcription factors or blocking signalling pathways controlling osteogenesis.^24^, ^27^, ^28^ But in this study, we found that, if ROS was eliminated by antioxidants, knockdown of EZH2 would not promote the osteogenic differentiation of BMSCs, suggesting that EZH2 suppressed the osteogenic differentiation of old BMSCs through exaggerating intracellular oxidative stress. Interestingly, EZH2 functions through distinct pathways in young and old BMSCs. Further analysis using chip‐on‐CHIP combining with bioinformatic analysis would be a possible technique to reveal the complicated mechanism of EZH2 to regulate stem cell ageing.

Foxo1 functions as a signalling integrator for homeostasis maintenance in stem cells.^10^ Accordingly, multiple pathways converge on Foxo1 to control the activity of FOXOs.^10^ In normal cells, high levels of ROS facilitate the translocation of Foxo1 into the nucleus, leading to increased transcriptional activity. However, this protective mechanism was disabled in senescent BMSCs. Here, we found that EZH2 epigenetically inhibited the transcription of Foxo1 by increasing H3K27me3 on its promoter in senescent BMSCs. This finding suggested that epigenetic regulators also directly control the transcription of Foxo1 to modulate its function. Since previous studies mainly focused on the posttranscriptional regulation of Foxo1, more attention is necessary to investigate the epigenetic regulation of the transcription of Foxo1.

To further understand the mechanism of epigenetic regulation on senescence of BMSCs, CHIP‐sequence data of EZH2 and H3K27me3 in young and old BMSCs might be valuable.

Since epigenetic aberrations are potentially reversible, the cellular senescence induced by epigenetic mechanisms is theoretically reversible. Rejuvenation of ageing by epigenetic therapy emerges as a hot field in the research of ageing. Here, we showed that inhibiting the function of EZH2 partly recovered the osteogenic capacity of BMSCs. Several small molecules that suppress the function of EZH2, including tazemetostat and DZNep, have been approved by FDA for the treatment of several diseases. But these drugs have not been tested in the treatment of osteoporosis. According to our findings, epigenetic therapy inhibiting the function of EZH2 might be a promising treatment to prevent osteoporosis in the aged population. It remains elusive whether inhibitors of EZH2 could surpass the effects of osteoanabolics teriparatide and abaloparatide. But the mechanism of EZH2 is totally different from that of traditional osteoanabolics, suggesting that these drugs might be applied together to achieve better outcomes in the clinic.

## CONFLICT OF INTEREST

The authors declare that the research was conducted in the absence of any commercial or financial relationships that could be construed as a potential conflict of interest.

## AUTHOR CONTRIBUTION

**Xiaoxia Su:** Conceptualization (equal); Data curation (lead); Formal analysis (equal); Funding acquisition (equal); Investigation (lead); Methodology (equal); Project administration (equal); Resources (lead); Validation (equal); Writing – original draft (equal). **Haoyu Zhang:** Data curation (equal); Formal analysis (equal). **Fengzhen Lei:** Data curation (supporting); Investigation (supporting); Methodology (supporting); Validation (supporting); Visualization (supporting). **Rui Wang:** Methodology (supporting); Software (supporting); Validation (supporting); Visualization (supporting). **Tingting Lin:** Methodology (supporting); Validation (supporting); Visualization (supporting). **Li Liao:** Conceptualization (lead); Data curation (equal); Formal analysis (equal); Funding acquisition (equal); Project administration (equal); Software (equal); Supervision (equal); Validation (equal); Visualization (equal); Writing – original draft (lead); Writing – review & editing (lead).

## Supporting information

Supplementary Material

## Data Availability

The data that support the findings of this study are available from the corresponding author upon reasonable request.
