# Supplementary material for: Epigenetic therapy attenuates oxidative stress in BMSCs during ageing
Source: J Cell Mol Med. 2021 Dec 7;26(2):375–84. doi: 10.1111/jcmm.17089 (PMC8743666; doi:10.1111/jcmm.17089)
Supplement: Supplementary file 1 — Supplementary Material [file JCMM-26-375-s001.docx]

**Supplementary Figure**


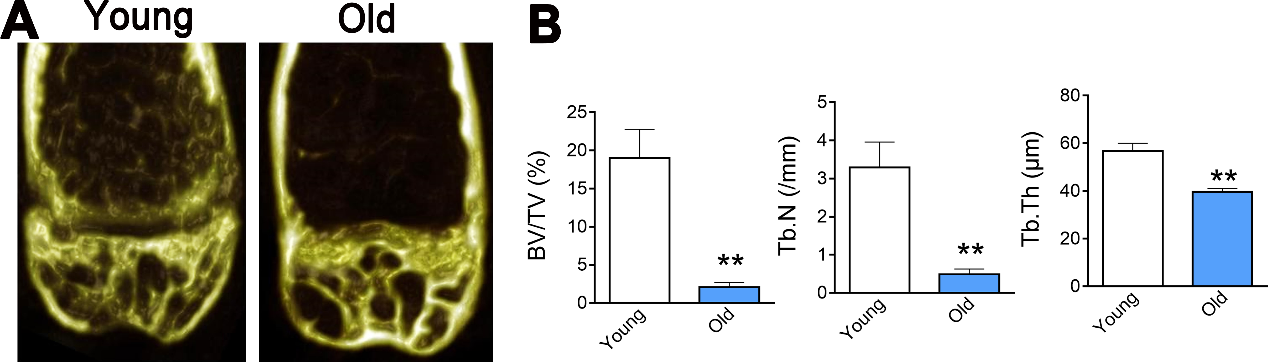


Supplementary Figure S1. Micro-CT analysis of femurs of young and old mice.

(A) Representative images of the bone structure of the metaphysis of distal femurs derived from 2-month old and 20-month old mice. (B) Morphological parameters of trabecular bone were analyzed. n=5. (*t*-test) **P<0.01.


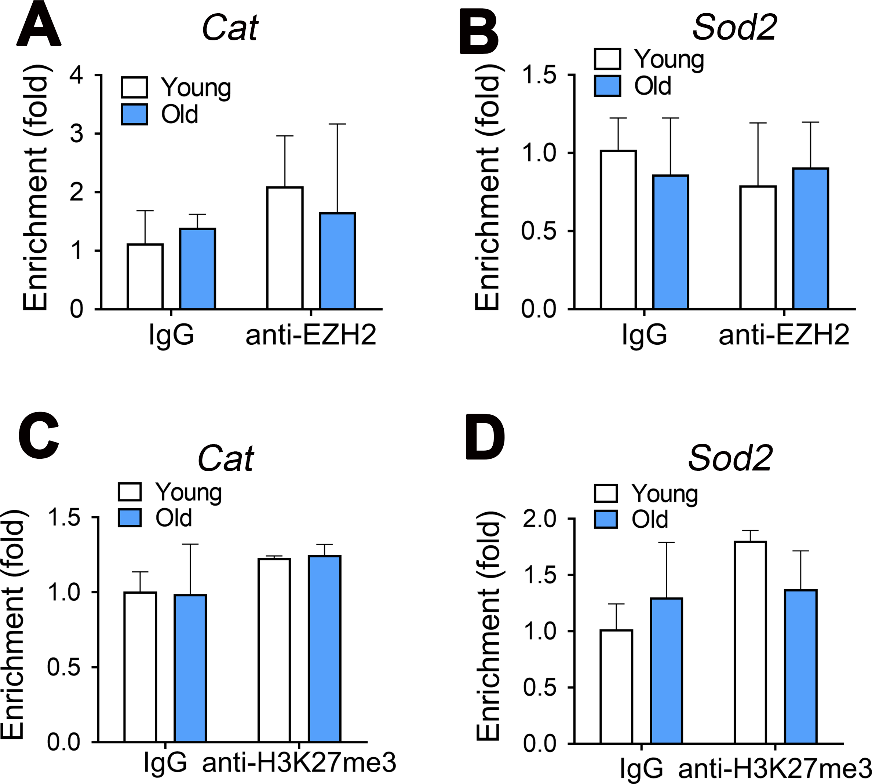


Supplementary Figure S2. CHIP analysis of the binding of EZH2 at the promoter region of Cat and Sod2 gene

(A) CHIP analysis of the binding of EZH2 at the promoter region (251~500bp upstream of protein-coding regions) of the *Cat* gene. n=4. (ANOVA) (B) CHIP analysis of the binding of EZH2 at the promoter region (251~500bp upstream of protein-coding regions) of the *Sod2* gene. n=4. (ANOVA) (C) CHIP analysis of the binding of H3K27me3 at the promoter region (251~500bp upstream of protein-coding regions) of the Cat gene. n=4. (ANOVA) (D) CHIP analysis of the binding of EZH2 at the promoter region (251~500bp upstream of protein-coding regions) of the Sod2 gene. n=4. (ANOVA)

**Supplementary Table**

**Supplemental Table S1** Real-time PCR primer pairs.

| **Gene** | **Species** | **Description** | **Primer sequence** |
| --- | --- | --- | --- |
| *β-actin* | *Mus musculus* | Forward | 5’-CTTCGGGAAGGAGCTCAAA-3’ |
|  |  | Reverse | 5’-TGCCTCGTTGTTGTGCAAG-3’ |
| *P16 ^INK4a^* | *Mus musculus* | Forward | 5'-CCAGACCGACGGGCATAG-3' |
|  |  | Reverse | 5'-CGCCTTCGCTCAGTTTCTCA -3' |
| *P53* | *Mus musculus* | Forward | 5'-ATGAACCGCCGACCTATCC-3' |
|  |  | Reverse | 5'-GGCAGGCACAAACACGAAC-3' |
| *Foxo1* | *Mus musculus* | Forward | 5'-ACGAGTGGATGGTGAAGAGC-3' |
|  |  | Reverse | 5'-TGCTGTGAAGGGACAGATTG-3' |
| *Sod2* | *Mus musculus* | Forward | 5'-CAGACCTGCCTTACGACTATGG-3' |
|  |  | Reverse | 5'-CTCGGTGGCGTTGAGATTGTT-3' |
| *Atm* | *Mus musculus* | Forward | 5'-GATCTGCTCATTTGCTGCCG-3' |
|  |  | Reverse | 5'-GTGTGGTGGCTGATACATTTGAT-3' |
| *Cat* | *Mus musculus* | Forward | 5'-AGCGACCAGATGAAGCAGTG-3' |
|  |  | Reverse | 5'-TCCGCTCTCTGTCAAAGTGTG-3' |
| *Runx2* | *Mus musculus* | Forward | 5’-CCGCAGGTCACTACCAGCCAC-3’ |
|  |  | Reverse | 5’-GAGGGCAGCACCGAGCACAG-3’ |
| *Ocn* | *Mus musculus* | Forward | 5’-CCTATTGGCCCTGGCCGCAC-3’ |
|  |  | Reverse | 5’-GACACCCTAGACCGGGCCGT-3’ |
